# KIF5A upregulation in hepatocellular carcinoma: A novel prognostic biomarker associated with unique tumor microenvironment status

**DOI:** 10.3389/fonc.2022.1071722

**Published:** 2023-01-06

**Authors:** Qi Liu, Yu-yang Liu, Xue-min Chen, Bing-yan Tao, Kuang Chen, Wei-min Li, Chang-tao Xu, Ying Shi, Hao Li, Hao-run Liu

**Affiliations:** ^1^ Faculty of Hepato-Pancreato-Biliary Surgery, The First Medical Center, Chinese PLA General Hospital, Beijing, China; ^2^ Department of Hepatobiliary, The Eighth Medical Center, Chinese PLA General Hospital, Beijing, China; ^3^ Medical School of Chinese PLA, Beijing, China; ^4^ Senior Department of Otolaryngology-Head & Neck Surgery, Chinese PLA General Hospital, Beijing, China; ^5^ School of Medicine, University of Electronic Science and Technology of China, Chengdu, China; ^6^ Department of Neurobiology, Beijing Institute of Basic Medical Sciences, Beijing, China

**Keywords:** KIF5A, prognostic biomarker, liver hepatocellular carcinoma, tumor microenvironment, immunotherapy

## Abstract

Liver hepatocellular carcinoma (LIHC) is one of the most common liver malignancies with high mortality and morbidity. Thus, it is crucial to identify potential biomarker that is capable of accurately predicting the prognosis and therapeutic response of LIHC. Kinesin family member 5A (KIF5A) is a microtubule-based motor protein involved in the transport of macromolecules such as organelle proteins in cells. Recent studies have illustrated that the high expression of KIF5A was related to poor prognosis of solid tumors, including bladder cancer, prostate cancer, and breast cancer. However, little is currently known concerning the clinical significance of KIF5A expression in LIHC. Herein, by adopting multi-omics bioinformatics analysis, we comprehensively uncovered the potential function and the predictive value of KIF5A in stratifying clinical features among patients with LIHC, for which a high KIF5A level predicted an unfavorable clinical outcome. Results from KIF5A-related network and enrichment analyses illustrated that KIF5A might involve in microtubule-based process, antigen processing and presentation of exogenous peptide antigen *via* MHC class II. Furthermore, immune infiltration and immune function analyses revealed upregulated KIF5A could predict a unique tumor microenvironment with more CD8+T cells and a higher level of anti-tumor immune response. Evidence provided by immunohistochemistry staining (IHC) further validated our findings at the protein level. Taken together, KIF5A might serve as a novel prognostic biomarker for predicting immunotherapy response and could be a potential target for anti-cancer strategies for LIHC.

## Introduction

According to global cancer statistics, the mortality rate of liver hepatocellular carcinoma (LIHC) ranks third with a steady upward trend among various cancer types, posing a serious threat to patients’ life and health (1). The World Health Organization estimates that, based on its annual forecasts, more than a million people will die from LIHC by 2030 (2). At present, the pathogenesis of LIHC has not been thoroughly determined, and the widely-accepted view is that coinfection of hepatitis C and hepatitis B virus with combination of other chemical and physical factors including excessive alcohol consumption, aflatoxin exposure, and smoking tobacco contribute to the formation of LIHC (3). Surgical resection is the most effective management for LIHC. Notwithstanding the wide clinical application of minimally invasive technology and laparoscopic surgery, the five-year recurrence rate of LIHC patients undergoing radical surgical resection is as high as 70% (4). Radiotherapy, transcatheter arterial chemoembolization (TACE), targeted therapy, and immunotherapy have also been attempted for LIHC treatment. However, the high recurrence rate, early distant metastasis and frequent resistance to chemotherapy and radiotherapy still result in poor prognosis of LIHC patients (5).

Immunotherapy has been attempted used in various cancer types such as melanoma (6), lung cancer (7), gastric cancer (8), breast cancer (9), head and neck squamous carcinoma (10), and acute lymphoblastic leukemia (11). Studies have shown that the immune microenvironment has a critical role in the pathogenesis of LIHC, where immune tolerance and evasion mechanisms play important roles (12, 13). Hence, immunotherapy, such as immune checkpoint inhibitors (ICI) based treatment, adoptive cell therapy (ACT), and genetically engineering vaccines, has emerged as a new anti-tumor therapy used for LIHC patients in recent years. The objective of immunotherapy is to kill tumor cells by activating immune effector cells *in vivo* or inhibit the occurrence and development of tumor cells by activating anti-tumor immune response, rather than directly killing or interfering with tumor cells, so as to prolong the survival time of patients. At present, sorafenib and lenvatinib have been used as first-line treatments for advanced LIHC (14), while regorafenib, cabozantinib, and ramucirumab can help to enhance therapeutic effects (15). The United States Federal Drug Administration (FDA) has also granted approval for the programed death-1 (PD-1) inhibitors nivolumab and pembrolizumab as second-line treatments for advanced LIHC (16). To our regret, only a fraction of LIHC patients are vulnerable and benefit from the treatment, the others are refractory to these agents during the progress of LIHC. Although a series of studies have been conducted to determine predictive biomarkers, LIHC-related immune therapy is still in its infancy. Basic research and clinical trials exploring immunotherapy biomarkers to predict LIHC treatment efficacy are still limited. Therefore, it is of great significance to find effective biomarkers for early detection of LIHC and stratification of patients who could benefit from immunotherapy.

Kinesin family member 5A (KIF5A) is a microtubule-based motor protein involved in the transport of macromolecules such as organelle proteins in cells (17), which could modulate the cell cycle including mitosis and meiosis, proliferation, and differentiation (18). KIF5A is involved in the transport of mitochondria and lysosomes to maintain nerve cell function, manifesting a significant role on maintaining neuronal homeostasis and function (19). Of note, the high expression of KIF5A was related to exacerbated prognosis of solid tumors, including bladder cancer (20), prostate cancer (21), and breast cancer (22). However, little is currently known concerning the clinical significance of KIF5A expression in LIHC.

Therefore, in this study, we aimed to explore the clinical significance of KIF5A in LIHC *via* bioinformatics analysis. We demonstrated that KIF5A is closely related to immune response, immune cells and immune microenvironment in LIHC. The overexpression of KIF5A is associated with poor clinic prognosis but might be a novel therapeutics target for immunotherapy.

## Materials and methods

### Dataset collection and normalization

The RNA-seq data and corresponding clinical information were retrieved from the TCGA database (https://portal.gdc.cancer.gov/). Prognostic information of LIHC patients was obtained from Liu et al. (23). Raw data were normalized with the transcripts per million (TPM) method, and log_2_ (TPM+1) transformation was also performed for subsequent investigation.

### KIF5A-related expression analysis

Pan-cancer analyses of KIF5A were constructed using the R software (v.3.6.3), with the “gglot2” package used for visualization. Additionally, the expression level of KIF5A was also investigated in LIHC patients with different clinical characteristics, including histological grade, vascular invasion, AFP level and overall survival (OS) event. Furthermore, The Human Protein Atlas (HPA) database (http://www.proteinatlas.org) was utilized to retrieve immunohistochemistry staining images of KIF5A, CD8a and PD-1 (24).

### Survival analysis

The LIHC cohort was categorized into two groups by median of KIF5A. Kaplan-Meier survival analysis was used to determine the association of KIF5A expression level with OS and disease specific survival (DSS) in LIHC patients. Subgroup investigation was also constructed to further evaluate the prognostic value of KIF5A in LIHC patients with different clinical characteristics. Additionally, KIF5A-related survival analyses based on different immune infiltration status were constructed using Kaplan-Meier Plotter database (25). The log-rank test was chosen to estimate the difference. Statistical analysis was performed *via* “survival” package and visualization was constructed by “survminer” package.

### Univariate and multivariate cox regression analysis

To determine whether the high expression level of KIF5A was an independent indicator to predict patients’ prognosis,Cox proportional hazard regression analyses were constructed on LIHC patients in TCGA database. Initially, univariate Cox regression analysis were conducted, and confounding features were chosen with p <0.1. Subsequently, multivariate Cox regression analysis were performed. Statistical analyses were completed by “survival” package.

### Construction of KIF5A-related network and functional enrichment

Gene-gene interaction (GGI) network was constructed by GeneMANIA (http://www.genemania.org) and functional enrichment of GGI network was completed by GeneMANIA automatically (26). Similarly, Protein-protein interaction (PPI) network and its potential biological functions were performed using STRING database (https://string-db.org/) automatically (27). To further evaluate whether KIF5A was associated with antigen presentation, the expression level of antigen presentation-related markers in different groups (KIF5A-high and low group) was also investigated. Antigen presentation-related markers were collected from Thorsson et al. (28).

### Gene set enrichment analysis

To deeply explore potential biological function of KIF5A in LIHC, Gene Set Enrichment Analysis (GSEA) was performed using the CAMOIP database (http://www.camoip.net/) (29). Briefly, the comparison of the GSEA was preformed between KIF5A-high and low group, and the results were directly downloaded from CAMOIP database.

### KIF5A-related mutational landscape in LIHC

Mutational landscape of LIHC was constructed by the CAMOIP database. Briefly, somatic mutations including frameshift del, frameshift ins, inframe del, inframe ins, missense mutation, nonsense mutation and splice site were compared in two groups (KIF5A-high and low group). The results were summarized in a heatmap which was generated by CAMPOIP automatically. Additionally, the expression level of KIF5A was also investigated between TP53 wild-type (WT) and TP53 mutated-type (MT).

### KIF5A-related tumor microenvironment analyses

To comprehensively illustrate KIF5A-related tumor microenvironment (TME), the CAMPOIP database was utilized to construct an integrated analysis, including immune infiltration (EPIC and MCPcounter methods), immune checkpoint molecules and immune scores (30–32). The comparison between KIF5A-high and low group were automatically completed by CAMPOIP and final results were directly downloaded from it.

### Immunohistochemistry and quantification

This research was approved by the Institutional Research Ethics Committee of the PLA General Hospital (No. S2021-608-02). Written informed consents were obtained from the patients or their legal guardians. Five paired formalin-fixed, paraffin-embedded LIHC tissues and corresponding adjacent tissues were used for immunohistochemistry (IHC) staining. Briefly, 4 mm tissue sections were mounted on glass microscope slides, deparaffinized in xylene, and then rehydrated in alcohol with increasing dilutions. Antigen retrieval was carried out in a water bath at a high temperature. Endogenous peroxidases were quenched with 3% hydrogen peroxide. Then, these sections were rinsed three times with PBS, incubated with calf serum for 10 minutes to block nonspecific antigens, incubated with anti-KIF5A polyclonal primary antibody (1:200, 21186-1-AP, Proteintech, China) overnight at 4°C, washed three times with PBS, and then incubated with secondary antibody for 20-30 minutes at room temperature (RT). Finally, these sections were observed by an optical microscope and three representative visual fields were collected for further assessment. IHC staining were evaluated by two independent pathologists who were blinded to the clinicopathological information. The intensity of staining was classified into the following categories: 0 (negative), 1 (weak), 2 (moderate) and 3 (strong). The percentage of positive staining cells was scored as follows: 0, no positive stained area; 1, <25%; 2, 25%–50%; and 3, >50%. IHC staining score was obtained by multiplying the intensity with the positive percentage, which yielded a result ranging from 0 to 12.

### Statistical analysis

For bioinformatics analyses, the Wilcoxon rank-sum was used to determine the statistical significance between two groups (KIF5A-high and low group). Associations between KIF5A expression and mutation frequencies were statistically evaluated by fisher’s exact test. All statistical analysis was performed using R software (v.3.6.3). To validate KIF5A expression in LIHC and paired adjacent tissues, normality was assessed with Shapiro–Wilks test and the paired Student’s t-test was used for two groups comparison. IHC staining score were analyzed using GraphPad Prism software (GraphPad Prism version 9.0.0 for Windows), and a two-tailed *p <*0.05 was considered significant.

## Results

### Elevated KIF5A expression in LIHC

**Figure 1:**
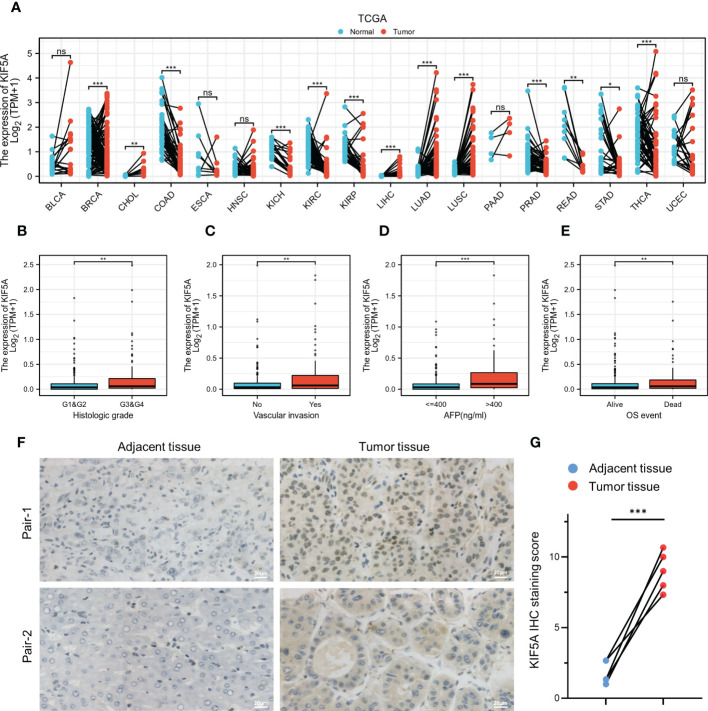
Elevated KIF5A expression in LIHC. **(A)** Pan-cancer analyses of KIF5A expression level based on TCGA database. **(B)** Correlation between KIF5A expression and histologic grade in LIHC. **(C)** Correlation between KIF5A expression and vascular invasion in LIHC. **(D)** Correlation between KIF5A expression and AFP level in LIHC. **(E)** Correlation between KIF5A expression and OS event in LIHC. **(F)** Representative IHC staining in LIHC and paired adjacent tissue. Scare bars, 20μm. **(G)** Analysis of IHC staining score of KIF5A in LIHC and paired adjacent tissue. Statistical significance was determined using the paired Student’s t-test. ***p < 0.001, **p < 0.01, *p < 0.05, *ns* not significant.

KIF5A expression was investigated across tumor types in the TCGA database using paired differential analyses. Results illustrated that a different expression level of KIF5A could be observed in neoplastic sites compared to that of the normal tissues, with the exception of bladder urothelial carcinoma (BLCA), head and neck squamous cell carcinoma (HNSC), esophageal carcinoma (ESCA), pancreatic adenocarcinoma (PAAD) and uterine corpus endometrial carcinoma (UCEC). Specifically, we identified a significantly elevated transcript level of KIF5A in LIHC in comparison with that of the paired-normal tissues (**Figure 1A**). To further explore the expression pattern of KIF5A in LIHC, we performed subgroup analyses by stratifying LIHC patients with different clinical features, including histological grade, vascular invasion, AFP level and OS event. Our data implied an upregulated KIF5A in patients with higher histologic grade (**Figure 1B**). In terms of vascular invasion status, the KIF5A level enhanced in LIHC with vascular invasion (**Figure 1C**). As for AFP level, the KIF5A expression was higher in LIHC with high level of AFP (>400 ng/ml) (**Figure 1D**). Additionally, the upregulation of KIF5A was also noted in LIHC with dead OS event (**Figure 1E**). Quantitative analysis of IHC staining score indicated that the protein level of KIF5A was obviously increased in LIHC tissues compared with paired adjacent tissues (*t* =9.33; *df* = 4; *p* =0.0007). (**Figures 1F, G**).

### Increased KIF5A expression is correlated with unfavorable prognosis

**Figure 2:**
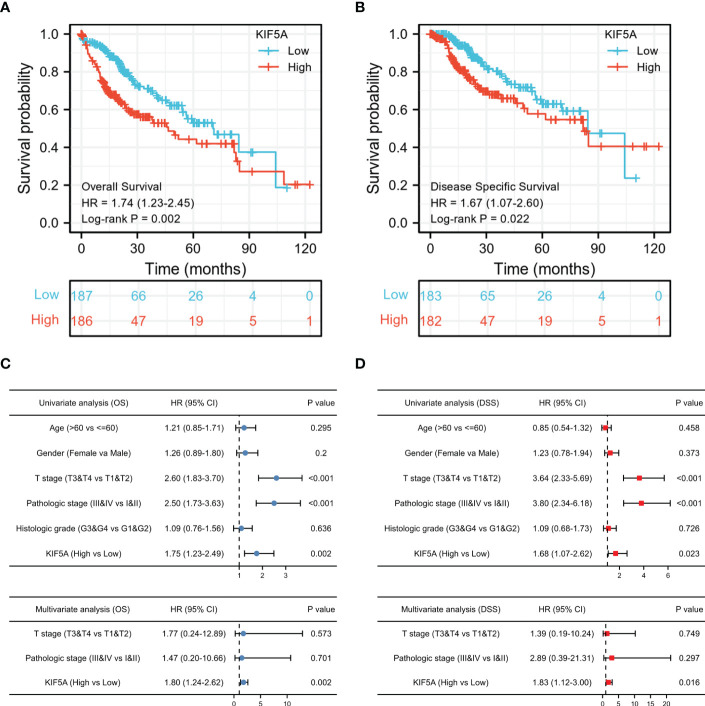
Increased KIF5A expression is Correlated with Unfavorable Prognosis. **(A)** Patients with upregulated KIF5A expression tended to have relatively lower OS (p= 0.002). **(B)** Patients with upregulated KIF5A expression tended to have relatively lower DSS (p= 0.022). **(C)** Univariate and multivariate Cox regression analysis of OS. **(D)** Univariate and multivariate Cox regression analysis of DSS.

Given that a high KIF5A expression level could potentially associated with malignant phenotype of LIHC, we constructed survival analyses to further evaluate the predictive value of KIF5A. According to Kaplan-Meier curves, LIHC patients with higher KIF5A expression had relatively lower OS [hazard ratio (HR) =1.74; 95% confidence interval (CI) =1.23-2.45; *p* =0.002] and DSS (HR =1.67; 95% CI =1.07-2.60; *p* =0.022) (**Figures 2A, B**). Univariate Cox regression analysis revealed that upregulated expression of KIF5A could predict unfavorable OS (HR =1.75; 95% CI =1.23-2.49; *p* =0.002) and DSS (HR =1.68; 95% CI =1.07-2.62; *p* =0.023). Additionally, multivariate Cox regression analysis illustrated that KIF5A might be an independent biomarker for predicting deteriorative prognosis including OS (HR =1.80; 95% CI =1.24-2.62; *p* =0.002) and DSS (HR =1.83; 95% CI =1.12-3.00; *p* =0.016) in LIHC patients (**Figures 2C, D**).

### Predictive value of the KIF5A based on subgroups analyses

**Figure 3:**
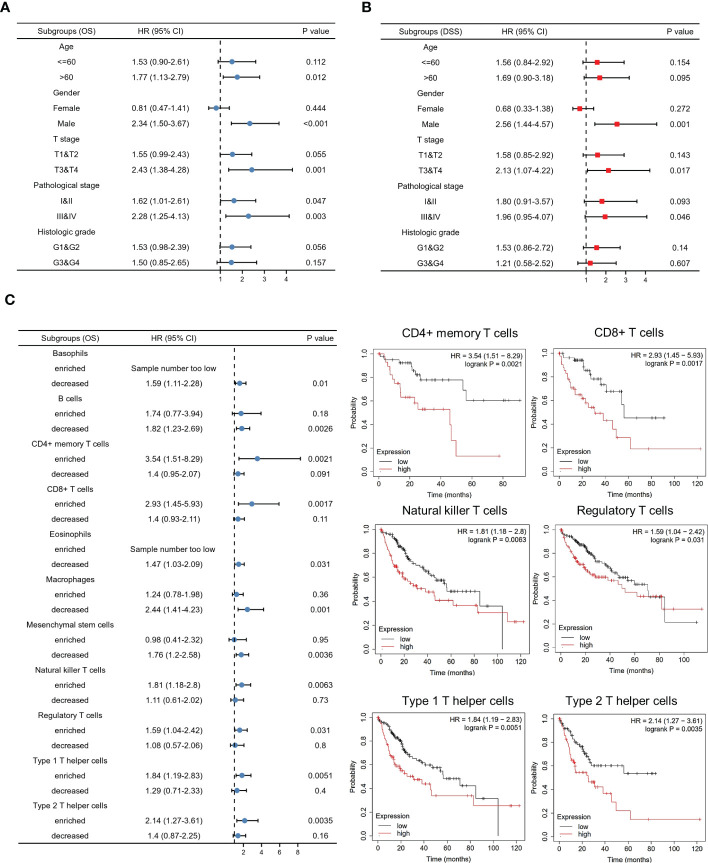
Predictive value of the KIF5A based on subgroups analyses. **(A)** Associations between KIF5A expression and the OS in different clinical subgroups of LIHC. **(B)** Associations between KIF5A expression and the DSS in different clinical subgroups of LIHC. **(C)** Associations between KIF5A expression and the OS in different immune infiltration subgroups of LIHC.

To further validate our findings, analyses of subgroups were constructed to investigate the correlation between KIF5A expression and prognosis of LIHC patients with various clinical characteristics. Our results showed that LIHC patients with a high expression level of KIF5A had a lower OS compared to those with a low KIF5A level, including the subgroup of age >60 (HR=1.77; 95% CI=1.13-2.79; *p*=0.012), male (HR=2.34; 95% CI=1.50-3.67; *p*<0.001), T3&T4 (HR=2.43; 95% CI=1.38-4.28; *p*=0.001) and pathological stage III&IV (HR=2.28; 95% CI=1.25-4.13; *p*=0.003) (**Figure 3A**). Furthermore, high level of KIF5A might associated with lower DSS, including subgroup of male (HR=2.56; 95% CI=1.44-4.57; *p*=0.001) and T3&T4 (HR=2.13; 95% CI=1.07-4.22; *p*=0.017) (**Figure 3B**). As for different immune infiltration status, KIF5A might be an biomarker predicting poor prognosis in various subgroups, including basophils decreased subgroup (HR=1.59; 95% CI=1.11-2,28; *p*=0.01), B cells decreased subgroup, (HR=1.82; 95% CI=1.23-2.69; *p*=0.0026), CD4^+^ memory T cells enriched subgroup, (HR=3.54; 95% CI=1.51-8.29; *p*=0.0021), CD8^+^ T cells enriched subgroup, (HR=2.93; 95% CI=1.45-5.93; *p*=0.0017), eosinophils decreased subgroup, (HR=1.47; 95% CI=1.03-2.09; *p*=0.031), macrophages decreased subgroup, (HR=2.44; 95% CI=1.41-4.23; *p*=0.001), mesenchymal stem cells decreased subgroup, (HR=1.76; 95% CI=1.2-2.58; *p*=0.0036), natural killer T cells enriched subgroup, (HR=1.81; 95% CI=1.18-2.8; *p*=0.0063), regulatory T cells enriched subgroup, (HR=1.59; 95% CI=1.04-2.42; *p*=0.031), type 1 T helper cells enriched subgroup and (HR=1.84; 95% CI=1.19-2.83; *p*=0.0051) and type 2 T helper cells enriched subgroup (HR=2.14; 95% CI=1.27-3.61; p=0.0035) (**Figure 3C**).

### Potential biological function of KIF5A

**Figure 4:**
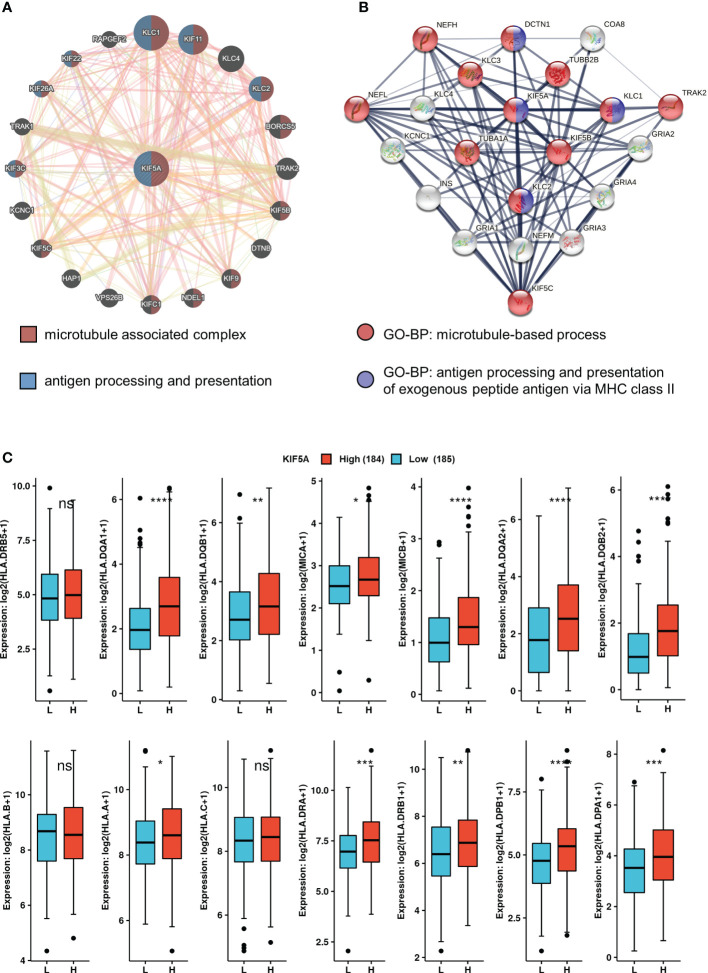
KIF5A-Related network and potential biological function. **(A)** GGI network of KIF5A and its potential biological functions. **(B)** PPI network of KIF5A and its potential biological functions. **(C)** Comparison of antigen presentation-related markers between two groups. ****p < 0.0001, ***p < 0.001, **p < 0.01, *p < 0.05, *ns* not significant.

To excavate the potential biological function of KIF5A, we firstly constructed GGI and PPI network. The GGI network analysis identified top 20 genes that interacted with KIF5A closely, including KLC1, KIF11, KLC4, KLC2,etc. Enrichment analysis revealed an association of these genes with microtubule associated complex and antigen processing and presentation (**Figure 4A**). Thereafter, the top 20 proteins binding with KIF5A were also screen. Correspondingly, enrichment analysis implied that KIF5A might involve in microtubule-based process and antigen processing and presentation of exogenous peptide antigen *via* MHC class II (**Figure 4B**). Hence, network analyses based on GGI, and PPI suggested that KIF5A might have an intimate relationship with the process of antigen processing and presentation. Further validation revealed that KIF5A-high group had significantly higher expression of most antigen presentation-related markers, except for HLA.DRB5, HLA.B and HLA.C (**Figure 4C**).

**Figure 5:**
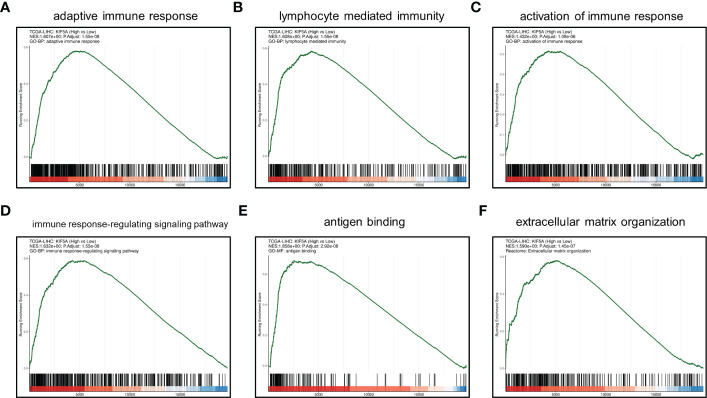
GSEA between KIF5A-high and low group. **(A)** adaptive immune response. **(B)** lymphocyte mediated immunity. **(C)** activation of immune response. **(D)** immune response-regulating signaling pathway. **(E)** antigen binding. **(F)** extracellular matrix organization.

GSEA manifested that upregulated KIF5A expression was associated with adaptive immune response, lymphocyte mediated immunity, activation of immune response, immune response-regulating signaling pathway, antigen binding and extracellular matrix organization (**Figures 5A–F**). Above results highlighted the latent functions of KIF5A in tumor immune response and TME, rendering us to deeply explore its biological role in subsequent analyses.

### Association between KIF5A and TP53

**Figure 6:**
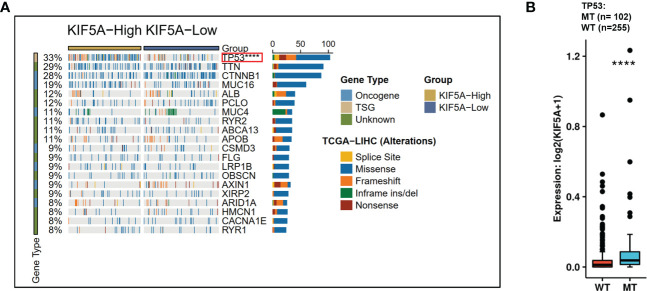
Correlations of KIF5A expression level with gene mutation. **(A)** KIF5A-Related Mutational Landscape in LIHC. **(B)** KIF5A expression level was positively correlated with the mutation frequency of *TP53*. ****p < 0.0001.

KIF5A-Related Mutational Landscape in LIHC revealed a significant higher frequency of TP53 mutations in KIF5A-high group (*p*< 0.0001) (**Figure 6A**). Additionally, significantly higher KIF5A expression levels were reported in the TP53-MT group than those in the TP53-WT group (*p*< 0.0001) (**Figure 6B**). These data suggest that high level of KIF5A might correlate with high mutant frequency of TP53.

### Upregulated KIF5A predicts unique TME in LIHC

**Figure 7:**
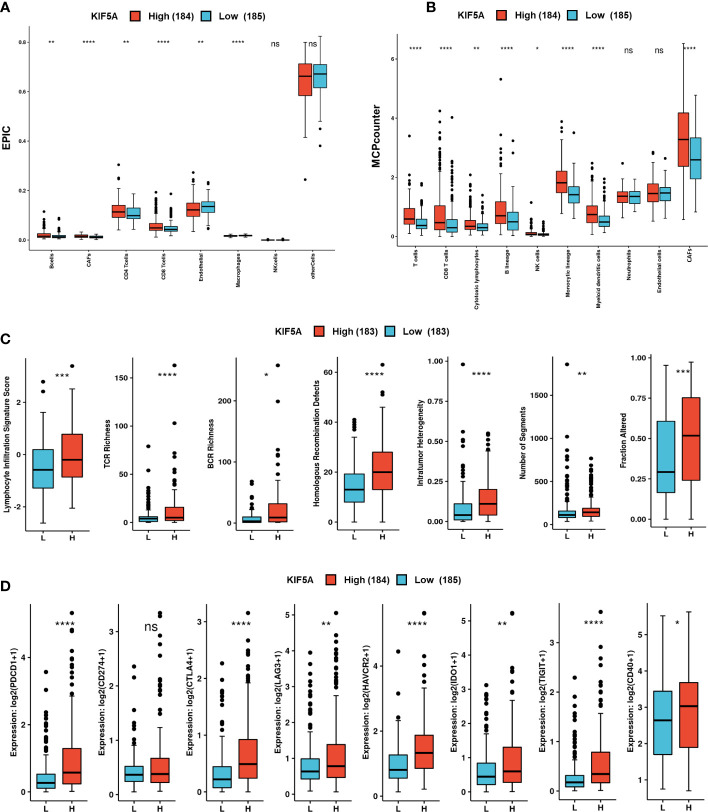
Upregulated KIF5A predicts unique TME in LIHC. **(A)** Immune infiltration analyses based on the EPIC method. **(B)** Immune infiltration analyses based on the MCPcounter method. **(C)** Comparison of immune scores between KIF5A-high and low group. **(D)** Comparison of immune checkpoint molecules between KIF5A-high and low group. ****p < 0.0001, ***p < 0.001, **p < 0.01, *p < 0.05, *ns* not significant.

Considering that the KIF5A was demonstrated to be involved in the procedure of immune response and extracellular matrix organization, we then probed the role of KIF5A in remodeling the TME. Immune infiltration analyses based on EPIC methods revealed that more B cells (*p* < 0.01), CAFs (*p* < 0.0001), CD4^+^ T cells (*p* < 0.01) and CD8^+^ T cells (*p* < 0.0001) were detected in KIF5A-high group (**Figure 7A**). Similarly, MCPcounter methods demonstrated that more T cells (*p* < 0.0001), CD8^+^ T cells (*p* < 0.0001), cytotoxic lymphocytes (*p* < 0.01), B lineage (*p* < 0.0001) and CAFs (*p* < 0.0001) infiltrated in KIF5A-high group (**Figure 7B**).

**Figure 8:**
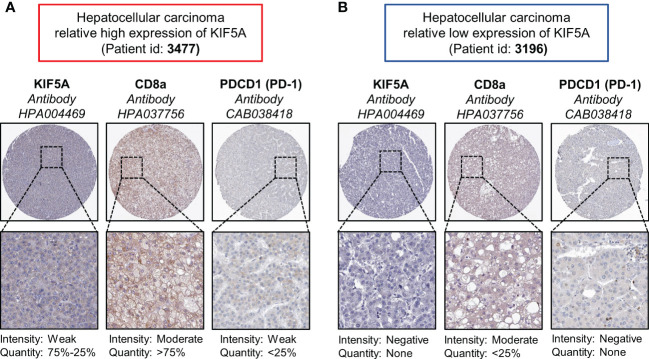
Associations between KIF5A with CD8a and PD-1 in LIHC. **(A)** Representative IHC staining pictures of LIHC with relative high expression of KIF5A. **(B)** Representative IHC staining pictures of LIHC with relative low expression of KIF5A.

Immune scores analyses revealed high levels of lymphocyte signature score (*p* < 0.001), TCR richness (*p* < 0.0001), BCR richness (*p* < 0.05), homologous recombination defects (*p* < 0.0001), intratumor heterogeneity (*p* < 0.0001), number of segments (*p* < 0.01) and faction altered (*p* < 0.001) (**Figure 7C**). Comparison of immune checkpoint molecules between two groups showed that higher levels of PDCD1 (p < 0.0001), CTLA4 (p < 0.0001), LAG3 (p < 0.01), HAVCR2 (p < 0.0001), IDO1 (p < 0.01), TIGHT (p < 0.0001) and CD40 (p < 0.05) were detected in KIF5A-high group (**Figure 7D**).Additionally, IHC staining showed that LIHC with relative high expression of KIF5A had higher expression (**Figure 8A**) levels of CD8a and PDCD1 in compared with LIHC with relative low expression of KIF5A (**Figure 8B**). All these data implied that upregulated KIF5A might be associated with a unique TME in LIHC.

## Discussion

The liver hepatocellular carcinoma (LIHC), which occurs in more than 90% of all primary hepatoma cases, ranks third in cancer-related deaths and is increasing rapidly (33). As a result, finding specific prognostic biomarkers to guide LIHC treatment and improve the overall survival of LIHC patients is extremely needed. In our current study, we constructed an integrated exploration on the relationship between KIF5A and LIHC. Expression analyses in pan-cancer revealed that KIF5A was abnormally expressed in various cancers, including LIHC (**Figure 1A**). Additionally, validation in clinical specimens using IHC staining further confirmed that KIF5A was abnormally upregulated in LIHC (**Figures 1F, G**). Expression of KIF5A in LIHC revealed that a high expression level of KIF5A might positively correlated with the malignant phenotypes of LIHC, suggesting that KIF5A might associated with LIHC disease progression (**Figures 1B–E**). Based on Kaplan-Meier curves, multivariate Cox regression analyses (**Figures 2A–D**) and subgroup analyses (**Figures 3A–C**), we uncovered that KIF5A might be a novel prognostic biomarker which could predict poor prognosis independently.

To illustrate the potential function of KIF5A in LIHC, we constructed KIF5A-related GGI and PPI network. Both enrichment analyses implied that KIF5A might involve in antigen processing and presentation (**Figures 4A, B**). Further validation revealed that KIF5A might positively correlated with various antigen presentation-related markers (**Figure 4C**). To elicit an effective anti-tumor immune response, antigen presentation needs to play an important role in the following two procedures (34). Firstly, antigens of cancer should be taken up by dendritic cells and presented to CD8^+^T cells. Secondly, CD8^+^T should recognize antigen which directly presented by tumor cells. Tumors could evade anti-tumor immune response *via* exploiting multiple escape mechanisms at both of these steps, including reduction of antigen presentation on their surface *via* downregulating surface expression of antigen presentation-related molecules (35). Thus, KIF5A-high LIHC with relative high level of antigen presentation-related markers might have upregulated antigen presentation process. These results prompted us to deeply explore the role of KIF5A in anti-tumor immune response and tumor microenvironment.

Similarly, GSEA revealed that upregulated KIF5A might be linked with adaptive immune response, lymphocyte mediated immunity, activation of immune response, immune response-regulating signaling pathway, antigen binding and extracellular matrix organization (**Figures 5A–F**). According to these results, KIF5A-high LIHC might have upregulated adaptive immune response mediated by lymphocyte. Immune infiltration analyses based on EPIC and MCPcounter methods demonstrated that KIF5A-high group have elevated CD8^+^T cells. Additionally, MCPcounter showed that more total T cells and cytotoxic lymphocytes in KIF5A-high group. Michele et al. illustrated that T-cells infiltration, especially CD8^+^ T cells, is a prerequisite for immune checkpoint blocking (36). Furthermore, higher densities of CD8+ T cells in pre-treatment biopsies can predict response to therapy (37). Thus, we can infer that KIF5A-high LIHC with more CD8+ T cells might be suitable for immunotherapy.

TP53 mutation were the most common gene alterations in LIHC (38). Numbers of studies have illustrated that the TP53 mutation were intimately associated with tumor immune microenvironment of LIHC. Wang et al. uncovered that TP53 mutations was related to higher tumor mutational burden which might predict better efficacy of immunotherapy in LIHC patients (39). Yang et al. illustrated that LIHC harbored TP53 neoantigen have higher cytotoxic lymphocytes infiltrations, CYT score and Immune score. Furthermore, they also suggested that TP53 neoantigen could regulate anti-tumor immunity and could be a novel target for immunotherapy in LIHC patients (40). Our study showed that high level of KIF5A might correlate with high mutant frequency of TP53 (**Figure 6**). According to these results, we can infer that KIF5A could also be an innovative biomarker reflecting unique tumor microenvironment of LIHC and need further exploration.

Based on CAMOIP database, we further explored the association between KIF5A expression with anti-tumor immune response. According to the analyses of immune scores, a high level of lymphocyte signature score, TCR richness and BCR richness were significantly correlated with high KIF5A expression in LIHC (**Figure 7C**). Thus, activation of multiple antitumor immune responses was associated with KIF5A expression in LIHC (28, 41). Given that tumor mutations can generate neoantigens which could subsequently prime a T-cell immune attack to tumor cells, we explored immune scores that related to neoantigens, including homologous recombination defects, intratumor heterogeneity, number of segments and faction altered (28, 42). We found that higher levels of these indicators were detected in KIF5A-high group (**Figure 7C**). These results pointed out LIHC with elevated KIF5A might have high levels of mutation burden and neoantigen load, so that these patients might be benefit from immunotherapy. Similarly, our data also demonstrated that high-KIF5A LIHC expressed significantly higher immune checkpoint molecules which might reflect more T cell infiltration and positive response to therapeutics of immune checkpoint inhibitors (**Figure 7D**) (28, 32). Above results suggested that for patients with high-level of KIF5A, although they had relatively worse prognosis, they might have a chance to elicit stronger anti-tumor response and get benefit from immunotherapy.

## Conclusion

In conclusion, this is the first report to reveal KIF5A as a novel biomarker for LIHC. We figure out latent biological function of KIF5A and its important role in LIHC tumor microenvironment. Furthermore, through multi-omics bioinformatics analysis, we identify that KIF5A might be an innovative indicator for predicting immunotherapy response. All these results proposed that KIF5A might be a potential target for anti-cancer strategies for LIHC.

## Data availability statement

The original contributions presented in the study are included in the article/supplementary material. Further inquiries can be directed to the corresponding author.

## Ethics statement

The studies involving human participants were reviewed and approved by the Institutional Research Ethics Committee of the PLA General Hospital. The patients/participants provided their written informed consent to participate in this study.

## Author contributions

QL and Y-YL conceived the bioinformatics analysis. YS and HL performed data collection. B-YT, KC and W-ML were responsible for data interpretation. QL, Y-YL and X-MC co-wrote the manuscript. C-TX and HL revised the manuscript. All authors contributed to the article and approved the submitted version.
